# Molecular evolution of the MAGUK family in metazoan genomes

**DOI:** 10.1186/1471-2148-7-129

**Published:** 2007-08-02

**Authors:** Aartjan JW te Velthuis, Jeroen F Admiraal, Christoph P Bagowski

**Affiliations:** 1 Department of Molecular and Cellular Biology, Institute of Biology, Leiden University, AL Leiden, 2333, The Netherlands; 2 Department of Integrative Zoology, Institute of Biology, Leiden University, AL Leiden, 2333, The Netherlands; 3 Sir William Dunn School of Pathology, University of Oxford, Oxford, OX1 3RE, UK

## Abstract

**Background:**

Development, differentiation and physiology of metazoans all depend on cell to cell communication and subsequent intracellular signal transduction. Often, these processes are orchestrated via sites of specialized cell-cell contact and involve receptors, adhesion molecules and scaffolding proteins. Several of these scaffolding proteins important for synaptic and cellular junctions belong to the large family of membrane-associated guanylate kinases (MAGUK). In order to elucidate the origin and the evolutionary history of the MAGUKs we investigated full-length cDNA, EST and genomic sequences of species in major phyla.

**Results:**

Our results indicate that at least four of the seven MAGUK subfamilies were present in early metazoan lineages, such as Porifera. We employed domain sequence and structure based methods to infer a model for the evolutionary history of the MAGUKs. Notably, the phylogenetic trees for the guanylate kinase (GK)-, the PDZ- and the SH3-domains all suggested a matching evolutionary model which was further supported by molecular modeling of the 3D structures of different GK domains. We found no MAGUK in plants, fungi or other unicellular organisms, which suggests that the MAGUK core structure originated early in metazoan history.

**Conclusion:**

In summary, we have characterized here the molecular and structural evolution of the large MAGUK family. Using the MAGUKs as an example, our results show that it is possible to derive a highly supported evolutionary model for important multidomain families by analyzing encoded protein domains. It further suggests that larger superfamilies encoded in the different genomes can be analyzed in a similar manner.

## Background

**Figure 1:**
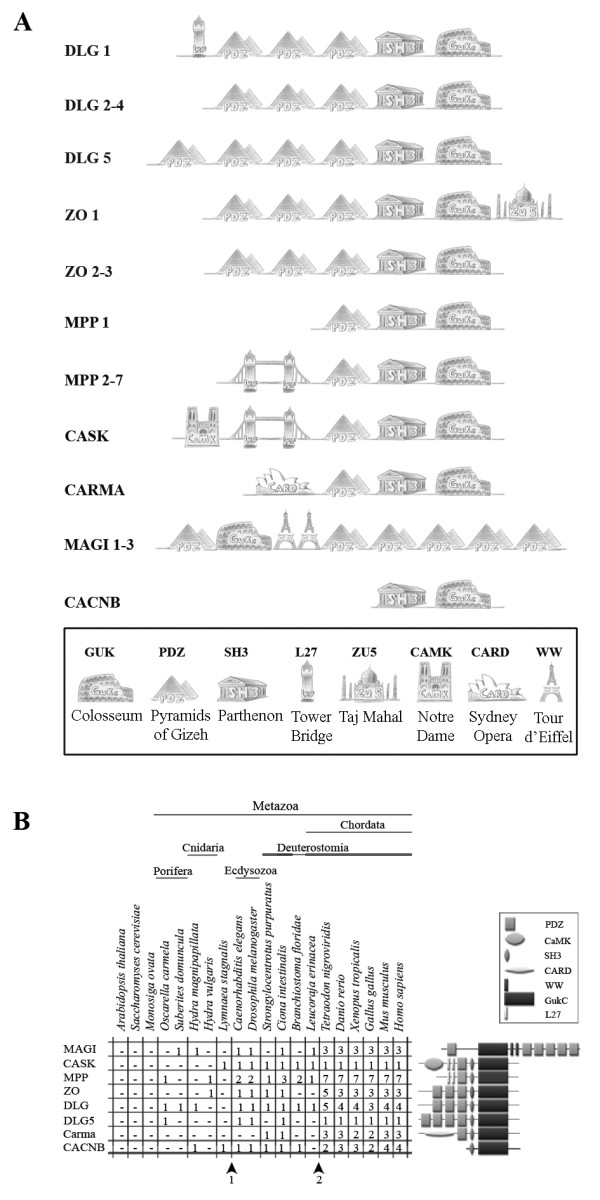
**Domain architectures of the MAGUK subfamilies and their distribution over the eukaryotic phyla**. (A) Eight subfamilies have been identified as members of the MAGUK family in our study. Depicted here in a general schematic representation are the domains present in these members. All members contain a central core comprised of a GK domain and several N and/or C-terminally positioned PDZ domains. All members except the MAGI proteins contain a SH3 domain that is nested between a PDZ and the GK domain. The CASK and MPP proteins contain N-terminal L27 domains, while *CARMA *genes encode CARD domains at this position. Names used are allowing a more systematic, but do not reflect in all cases the commonly used names e.g. DLG4 is better known as PSD95. A list of synonyms is given in table 1. The CACNB subfamily, commonly not regarded as a canonical MAGUK subfamily, only contains the SH3 and GK domains. Phylogenetic analysis presented here however shows that it is a related subfamily. (B) The MAGUK subfamily distribution over the eukaryotic phyla shows no homologs were found for Choanozoans/Protozoans, Plantae and Fungi, here represented by *Monosiga ovata*, *Arabidopsis thaliana *and *Saccharomyces cerevisae*, respectively. *Tetraodon nigroviridis *has duplicated DLG4, ZO1 and ZO2 encoding genes, whereas *Gallus gallus *lacked the gene for DLG4. The arrowheads on the bottom indicate two possible gene duplication events.

The membrane-associated guanylate kinase (MAGUK) family in mammals consists of 22 members, which vary in size and domain organization (Fig. 1A, Table 1). Despite these variances, the MAGUKs share a well conserved core structure, which is comprised of one or multiple PDZ domains, a Src homology 3 (SH3) and a guanylate kinase (GK) domain. The SH3 and GK domains surpass their canonical counterparts by interacting with each other to form a super domain. This characteristic is also remnant of voltage-gated calcium channel beta subunits that lack PDZ domains. The large MAGUK family, as already implied by their assortment of different gene architectures, encodes for a heterologous group of proteins with very diverse biological functions (for review see [1]).

Molecular studies essentially have established a wide variety of cellular functions for different MAGUKs. Examples include regulation of cellular processes including such as: establishment of cell polarity, tight junction formation, cell proliferation or apoptosis, cell differentiation and neuronal synapse transmission [2-5]. Mutations or changes in expression have often been found to cause defects in cell-cell adhesion, cell polarity, cell proliferation and subsequently development [6-9].

The domain architecture of the MAGUKs enables interaction with receptors, the actin-cytoskeleton and ion channels, but also allows for tethering of different MAGUK subfamily proteins together [10-12]. MAGUK proteins may contain up to six PDZ domains, two L27 and two WW domains (Fig. 1A). In addition to these domains, all MAGUKS, expect for the MAGI subfamily, contain a SH3 domain. The GK domain of the MAGUKs shares homology with *Guk1*of yeast but appears to have lost GMP binding capacity and catalytic activity [13]. Additionally, the SH3- and GK domain form an intermolecular interaction which renders the GK domain catalytic inactive and can function as a separate binding interface [14,15]. The understanding of the biochemical role and regulation of this super domain is limited, but it is intriguing that a similar SH3-HOOK-GK motif is present in voltage-gated calcium channels beta subunits [11,16].

Structural and molecular studies have shown that PDZ domains are pivotal features of scaffolding proteins and localize MAGUKs and their interaction partners to specialized membrane domains of neuronal and epithelial cells [1,2,17]. PDZ domains have a compact and modular structure, and allow MAGUK proteins to bind to C-terminal recognition sequences. Although originally identified in metazoans, the domain has been found to be spread through bacterial, fungi and plant lineages as well [1,18].

To date, phylogenetic analyses have been carried out only for individual members, such as ZO1 and the MAGIs [19,20]. Through our extensive phylogenetic analysis of the entire MAGUK family presented here, we were able to divide the MAGUKs into 7 subfamilies and to infer a probable evolutionary sequence of events that gave rise to the MAGUK domain architectures. We used a domain-by-domain analysis in order to determine that the PDZ-SH3-GK structure evolved only once in the course of evolution. In addition, we confirm our phylogenetic data by molecular modeling and provide evidence for the hypothesis that the MAGUK GK domain originated from a catalytically active GK domain and gradually lost its enzymatic characteristics when new subfamilies emerged.

## Results

### Taxonomic distribution and phyla-specific architectures

Many important biological roles have been described for members of the MAGUK family (reviewed in [1]). However, only very limited information is available about their evolutionary history. To analyze the phylogeny and molecular evolution of the MAGUKs, we initially gathered protein sequences for species of all major metazoan phyla, ranging from Porifera to Chordata, by using human, fruit fly, sponge and hydrozoan sequences as seed. Some sequences were readily available in GenBank and in helpful automated databases like Pfam (with many redundant sequences present) and Superfamily [21]. Many sequences were assembled from ESTs and genomic data. Sequences for all functional domains were identified (described in Methods) and categorized (see Additional file 1 for complete list).

No MAGUK homologs or MAGUK-like structures, represented by combinations of a GK domain with a SH3 and/or PDZ domains were found in protozoans, fungi and in plants. Our data shows that most canonical MAGUK family members are present throughout all animal phyla investigated (Fig. 1B). Basal metazoans, represented here by the sponges, *Oscarella carmela *and *Suberites domuncula*, and the cnidarians, *Hydra vulgaris *and *Hydra magnipapillata*, encode for several different MAGUK subfamily members (Fig. 1B). This was previously recognized when a MAGI homolog was characterized in *S. domuncula *[20] and a ZO member was described for *H. vulgaris *[19]. Here, we are able to add three new members to this list in basal metazoans, which now includes homologs of MPP, DLG and a DLG5 encoding genes (Fig. 1B and Additional file 1).

The protein architectures, with respect to the domain combinations, appear largely consistent and conserved throughout all metazoan phyla investigated. However, some lineage-specific differences can be found for example the *C. elegans dlg5 *gene, lacks the sequences for the GK-, the SH3- and the fourth PDZ-domain. The *M. musculus *and *H. sapiens Dlg5 *genes show variations as well, since they encode additional DUF622 or CARD domains (Q3UGX5, NP_004738). These domains are not present in all other species investigated.

We observed several species specific gene duplications within the MAGUK family for example several additional ZO homologs are present in the *T. nigroviridis *genome, compared to other vertebrates. A Tetraodon *Zo1 *gene can be found on both chromosome 5 and 13 and duplicated sequences for ZO-2 are positioned on the same two chromosomes (see Additional file 1). Gene duplications in teleosts have been described for the hox clusters [22,23].

### Phylogenetic analysis of guanylate kinase domains

**Figure 2:**
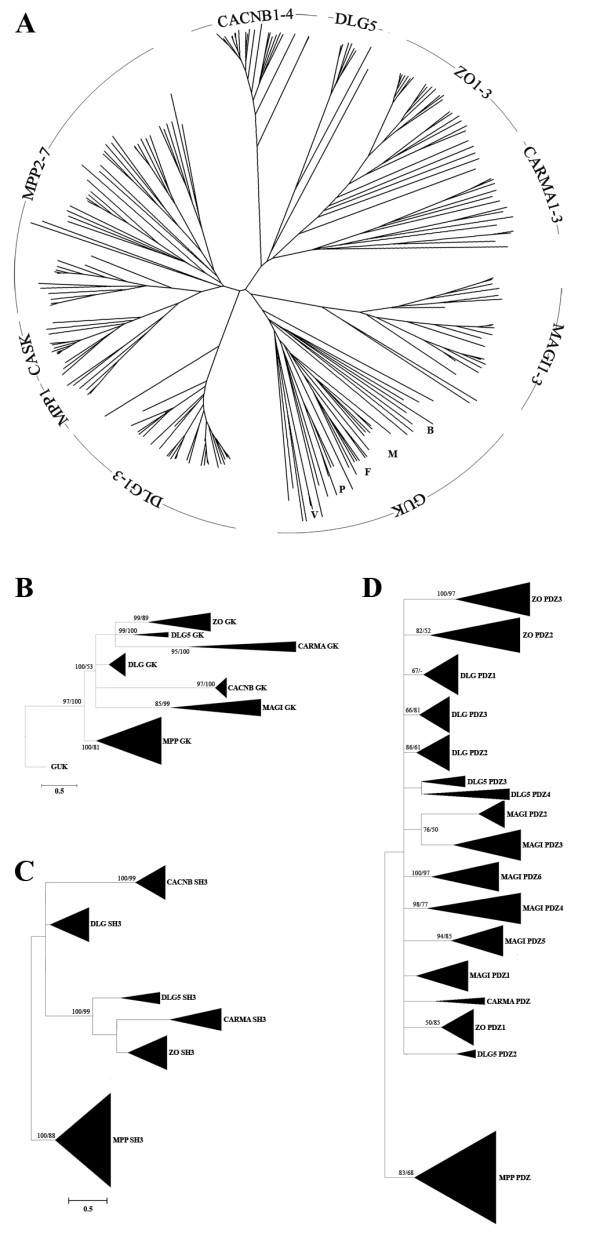
**Guanylate kinase dendrogram and summarized phylogenetic analyses**. (A) The dendrogram is based on all protein families currently known to contain GK sequences (note: this is not a phylogeny). These include the three families of the voltage-gated calcium channel beta subunit (CAB), the homologs of Guanylate kinase (GUK) and the MAGUK family. The core structure of the GUK is GK only, while the CAB and MAGUK families have a SH3-GK or PDZ-SH3-GK architecture respectively. The MAGUK and CAB families were only found in metazoan species, while sequences of GUK family members appeared dispersed over all eukaryotic lineages. In the GUK clade the metazoans are indicated with the letter M, the Fungi with F, Bacteria with B, Viruses with V and the Plants with P. Species that were included in this dendrogram as well as sequences used are listed in Additional file 2. (B-C) Summarized phylogenetic trees based on Bayesian consensus trees (see Additional file 3, 4, 5) for the GK, SH3 and PDZ domains, respectively. Numbers indicate % Bayesian posterior probability and % bootstrap Maximum Likelihood.

To elucidate the evolution of the MAGUK family genes and their gene architectures, we performed an analysis of the centrally positioned approximately 250 amino acid long GK domain. It has been suggested that the MAGUK GK domain evolved from other, enzymatically active, GK domains. The latter include domains like those present in the homologs of the *S. cerevisae guk1 *gene, which shares approximately 40% homology at the protein level [1]. Enzymatic GK domains can be found in most eukaryotic phyla, including plants, fungi, Protozoa and Metazoa, and are encoded by guanlylate kinase homologs (syn. Guk or GMP). The domains are also present in bacteria and certain viruses [e.g. [24]]. Our assembly (Fig. 2A) illustrates that the MAGUK protein subfamilies form a specific clustering pattern. Of particular interest is the clustering of the CASK subfamily with the MPP subfamily and very close to MPP1. Indeed, the MPP and CASK subfamilies are the only proteins in the MAGUK family that share an architecture containing two L27 domains (see Fig. 1A) [25]. The Guk and CACNB sequences clustered in separate clades.

On the basis of the aligned GK sequences that we used to construct the dendrogram we generated a phylogenetic tree (Fig. 2B; Additional file 3). Guk family sequences (not belonging to the MAGUKs) were used to root the tree. Judging from the GK phylogenetic relationships, the GK tree suggests that the MPP subfamily split of first from an active GK precursor closely followed by the MAGI and the CACNB subfamilies.

The phylogenetic analysis of the SH3 domains (Fig. 2C; Additional file 4), which may be 60–70 amino acids in length, showed the same basic evolutionary relationships as the GK domain-based phylogeny, which suggest that the two domains co-evolved along the same path. It must be noted that the MAGI subfamily is not present in the SH3 phylogeny since its members do not contain a SH3 domain.

### Comparison of GK 3D structures

**Figure 3:**
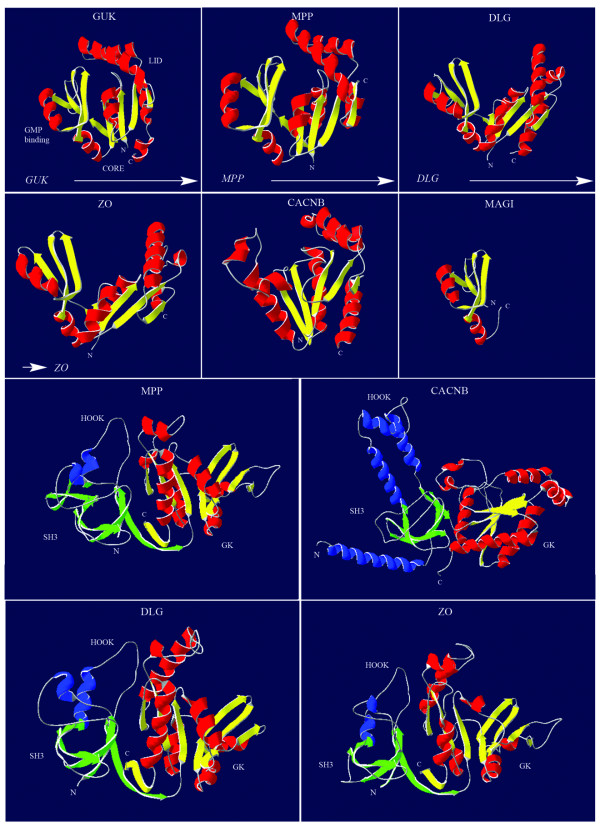
**Molecular modeling of guanylate kinase domains based on the human sequences**. Indicated in the left, uppermost panel are the three dynamic domains of the GMP guanylate Kinase: the GMP binding sub-domain, the CORE sub-domain and the LID sub-domain. The other panels show modeled structures of the MAGUK subfamilies, superimposed by Swiss Model. Arrows indicate the likely course of the structural evolution. The relationships established through our phylogenetic analysis are reflected here in the comparison of structures for the human representatives. The MPP structure still has a resemblance close to the human GMP guanylate Kinase, having a similar orientation of the three sub-domains of the GMP GK. The DLG and ZO subfamilies are also very closely related, when the presented 3D structures are compared. The MAGI GK could only be partially superimposed on known structures, sharing at least the GK GMP binding domain with the other MAGUK subfamilies. The used PDB files are listed in Additional file 2. The lowest four panels show representative models of the GK-HOOK-SH3 super domains of the MAGUK subfamilies MPP, DLG, ZO and CACNB. As in the upper panels the GK domain is colored yellow and magenta (β-strands and helices respectively), while the SH3 and HOOK regions are colored green and blue.

To substantiate our above described phylogeny, we created models of the GK and the super domain SH3-HOOK-GK, which is present in each MAGUK subfamily member, except the MAGIs. Canonical GK domains catalyze the reversible phosphoryl transfer from ATP to GMP [e.g. [25]]. Like other NMP kinases, GK proteins contain three essential, dynamic regions: the CORE domain, the LID domain and NMP-binding pocket (Fig. 3) [26]. Unique for the GK is that its GMP-binding domain is comprised of four β-sheets and a short helix, whereas others are purely α-helical [27].

Consistent with our phylogenetic analysis, it is apparent that the MPPs show the most extensive structural similarity with the active GK. The other subfamilies show less structural resemblance, but it is clear that the DLG and ZO subfamilies are more related to one another, than to the other subfamilies. This was also apparent from our phylogeny.

Indicated in the lowest two panels of figure 3, the SH3-HOOK-GK structures of representatives of the MPP and CACNB subfamilies are shown. Members of the DLG and ZO subfamilies adopted a structure largely similar to the MPP model, which places the CACNB subfamily by itself. This may be explained by the different biological role that CACNB is involved in, which requires a different structural organization compared to the scaffolding role of the canonical MAGUKs. The CARMA subfamily GK or SH3-HOOK-GK sequences could not be superimposed on any annotated structure in de database used. The PDB files and sequences used for the superimpositions, as well as their expect values, are listed in Additional file 2.

### Insertion of the WW domains in the MAGI subfamily

The MAGI proteins are different from the other MAGUK members as they lack a SH3 domain between the core GK and PDZ domains. They contain, however, two WW domains (Fig. 1A), which have been suggested to function in a similar fashion as the MAGUK SH3 domain, i.e. facilitating oligomerization [1,28]. From sequence alignments and annotation of the protein-protein interaction domains on these alignments we observed overlapping regions of the GK domain range and WW domains in the sequences of invertebrate species (in SMART, not shown). These overlaps were not present in the mammalian sequences, most likely because a larger, not well conserved, amino acid stretch was present between the two domains. Our alignment of the MAGI sequences revealed that there is a partial conservation of the proper GK domain (approx. 80 residues, compared to the normal 250) and indeed this area could also be reliably modeled (Fig. 3). These observations suggest that early in metazoan evolution, the two WW domains inserted into the C-terminal part of the MAGI GK domain, which thereafter resulted in a loss of this part of the domain.

### Diversification of the MAGUK architecture

We assumed that a thorough analysis of the PDZ domain (approx. 70–100 amino acids) would provide more insight into the architectural evolution of the MAGUK family. All C-terminally positioned PDZs of the DLG1-3 subfamily, the ZO1-3 subfamily and DLG5 are intimately related (Fig. 2D; Additional file 5). Within the DLG1-3 subfamily the three PDZ domains in these proteins are also clustered together very closely, which suggests rapid domain duplications. In the DLG5 subfamily, the fourth PDZ domain seems to have arisen from a domain duplication of the third DLG5 PDZ domain.

The first MAGI PDZ is closely related to the PDZs of the ZO, DLG subfamilies, which was expected from the GK phylogenetic tree. Interestingly however, all other MAGI PDZs (PDZs 2–6) are more related to one another and the MPP/CASK then to the most N-terminally positioned PDZ. These results suggest that the MAGI core structure is not due to an inversion of the GK domain as commonly assumed and reflected in the name (MAGI: membrane-associated guanylate kinase with an inverted arrangement of protein-protein interaction domains [28]).

## Discussion

The goal of the present study was to gain insight into the general and structural evolution of the MAGUK family. We further wanted to prove that it is possible to derive a supported evolutionary model by analyzing the phylogeny of individual domains found in multidomain protein families, like the MAGUKs. In order to map the general evolutionary history of this gene family we assembled a large dataset of sequences for all major metazoan phyla, and phylogenetically and structurally analyzed the core domains present.

Choanoflagellates may be closely related to metazoans and based on phylogenetic analyses and the observation that these protists have a collar of feeding tentacles reminiscent of sponge feeding cells, both had been suggested to form a monophyletic group called the Opisthokonta, [*e.g. *[24,30-32]]. Other protozoan species like *Giardia intestinalis *(syn. *G. lamblia*) were also suggested to be close to basal metazoans [33-35]. We attempted to identify MAGUK proteins and closely related structural homologs, which are vital for metazoan processes such as cell to cell communication, in these species as well, however, none could be found in protozoans. In addition, MAGUK sequences were not found in the genomes of bacteria, fungi and plant species. This suggests that the formation of the MAGUK structure, with its characteristic and centrally-positioned, non-functioning GK domain is essentially of metazoan origin and we speculate that the MAGUKs initially played important roles in cell to cell communication. The absence in Plantae can be explained by the late evolution of the MAGUKs, but it is tempting to speculate that cell to cell communication in plants is fundamentally different due to additional cell walls and thus might require different (scaffolding) modules than the MAGUKs.

Our search for MAGUK homologs has however, let to the identification of three new members in the most basal metazoans, which now includes homologs of MPP, DLG and a DLG5 encoding genes (Fig. 1B and Additional file 1). These findings imply that all canonical MAGUK family members arose very early during metazoan evolution. The CARMA genes are a likely exception and no homologs were identified in species more basal than the Deuterostomia. It is important to note that while this is a reasonable assumption at this time, not all genome projects have yet been completed. Thus, after completion they should be revisited.

We initiated our phylogenetic analysis with the GK and SH3 domain, and compared their evolutionary histories as they are both present in most MAGUK subfamilies. Indeed, the GK and SH3 domain show a similar phylogeny (Fig 2B and 2C; Additional file 3 and 4). Furthermore, these findings are supported by molecular modeling of the 3 dimensional structures of the GK domain and not contradicted by the phylogenetic analysis of the PDZ domains (Fig 2D; Additional file 5). This is an important finding, showing that the analysis of the independent domains of different sizes renders a similar evolutionary scenario.

In regard to the MAGUK family, we found that the Ca^2+ ^channel beta-subunit family seems to have evolved together with the MAGUK family. The CACNBs are commonly not considered to belong to the MAGUKs, but our phylogenetic results show that they are related, share a common ancestor and may thus represent a MAGUK subfamily.

Our analysis of the PDZ domains showed a bifurcated evolution (Additional file 5), with the MAGI PDZs linked to both groups (first PDZ to DLG, ZO and CARMA; the second to sixth PDZ to the MPPs). Additionally, we describe here that the WW domains likely inserted into the MAGI GK domain and then moved toward the C-terminus, leaving the GK domain deprived of its essential CORE and LID domain. At present, we do not have a good explanation for these events, but our findings are evidence against a complete inversion of the MAGI structure as was suggested earlier [28].

**Figure 4:**
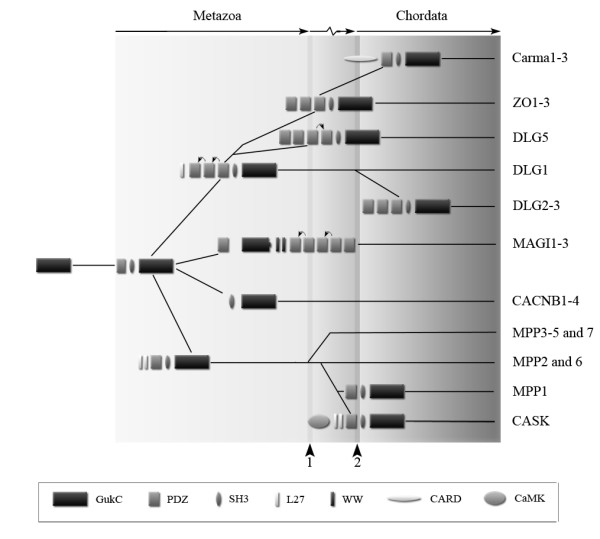
**Evolutionary model of the MAGUK subfamily-structure evolution**. Evolving from a catalytically active GK encoding gene, the family arose by obtaining both a PDZ and SH3 domain. Then the MPP subfamily branched off, assimilating L27 domains and, in the case of CASK, also a kinase domain. The MPP subfamily member known as MPP1 evolved last and lost its L27 domains. The CACNB and MAGI subfamilies arose through domain loss of the PDZ and SH3 domains, respectively. The later one also obtained WW domains, most likely resulting in a C-terminal truncation of the GK domain. The MAGUK core structure, consisting of a PDZ, SH3 and GK domain evolved further and gave rise, after duplication of the most C-terminal PDZ domain, to the DLG, ZO and, lastly, the CARMA subfamily. This PDZ domain duplicated another time at the birth of the DLG5 structure. The arrowheads on the bottom indicate two different gene duplication events. Arrows on top of the protein structures show PDZ domain duplication events derived from our phylogeny.

Based on our domain-by-domain analysis we propose a model to describe the structural evolution of the MAGUK family, including the CACNB family (Fig. 4). Evolving from an enzymatically active GK encoding gene, the family arose by obtaining both a PDZ and SH3 domain. Then the MPP subfamily split off, taking up L27 domains. The CACNB and MAGI subfamilies arose through domain loss of the PDZ and SH3 domains, respectively. The MAGUK core structure, consisting of a PDZ, SH3 and GK domain evolved further and gave rise, after duplication of the N-terminal PDZ domain, to the DLG, ZO and, lastly, the CARMA subfamily. Duplications of PDZ domains happened twice during evolution of the MAGUKs and are illustrated in our model (Fig. 4, arrows on top of protein structures).

In summary, we have derived here a highly supported evolutionary model for the MAGUK family by analyzing the phylogeny of individual domains. The results of our analysis provide strong evidence that other complex multidomain families and also larger superfamilies can be investigated in a similar way. Additionally, we provide evidence that places the Calcium-channel beta-subunit proteins within the MAGUK family from an evolutionary perspective.

## Conclusion

To elucidate the origin and the evolutionary history of the MAGUK family, we investigated full-length cDNA, EST and genomic sequences of species in major phyla. These data indicated that MAGUKs are present only in metazoan species and not encoded in protozoans, bacteria or plants. Phylogenetic analysis of our sequence data showed a matching evolutionary history for the central protein interaction domains of the MAGUKs. Supported further by structural evidence, we postulate that the MAGUKs evolved first as a GK-SH3 structure from an active GK enzyme, which is present in protozoa and plants. Then the PDZ domain was added to this structure, thereby completing the MAGUK core structure. New domains were subsequently added or duplications of the PDZ were made in order to give rise to the MAGUK assortment now present in vertebrates. Additionally, we provide evidence that places the Calcium-channel beta-subunit proteins within the MAGUK family, based on the evolutionary perspective of our research. Our results show that it is possible to derive a supported evolutionary model for important multidomain families by analyzing encoded protein domains. We suggest here that larger superfamilies can be analyzed in a similar manner.

## Methods

### Creating a functional domain database

Initially, a dataset of the functional domains found in the MAGUK family was established. For this purpose human homologs were searched with the Blastp algorithm [36] against the protein databases and with tBlastn against genome and EST databases of Ensembl, National Centre for Biotechnology Information and the Joint Genome Institute. Blast hits with high enough E-values were further analyzed with the protein domain prediction program SMART [37,38] and reblasted with Blastp for confirmation. Metazoan species searched for homologs were: *Branchiostoma floridae *(Bf), *Danio rerio *(Dr), *Caenorhabditis elegans *(Ce), *Ciona intestinalis *(Ci), *Drosophila melanogaster *(Dm), *Gallus gallus *(Gg), *Homo sapiens *(Hs), *Hydra magnipapillata *(Hm), *Hydra vulgaris *(Hv), *Mus musculus *(Mm), *Leucoraja erinacea *(Le), *Lymnaea stagnalis *(Ls), *Oscarella carmela *(Oc), *Strongylocentrotus purpuratus *(Sp), *Suberites domuncula *(Sd), *Tetraodon nigroviridis *(Tn) and *Xenopus tropicalis *(Xt).

### Alignment and phylogenetic analysis

Alignments were performed using ClustalX [39] with default parameter values, and manually refined in GeneDoc. The alignments were used for phylogenetic analysis employing both Bayesian analysis and Maximum Likelihood (ML). Bayesian trees were generated with MrBayes [40]. Rate variation across sites was modeled with a four rate gamma distribution and invariant sites, while the MCMC search itself was continued for 1,000,000 generations, sampled every 100 generations, and 2500 trees were discarded as burnin. The amino acid substitution model was set to mixed in order to reduce assumptions prior to analysis. For ML, alignments were bootstrapped 1000 times with the program Seqboot from the Phylip package [41]. Subsequently, phylogenetic trees were generated with the ML algorithm implemented in PhyML [42], with the amino acid substitution set at Jones-Taylor-Thornton. Other PhyML parameters were gamma distribution with four classes for across-site rate variation and optimization of the alpha parameter that was used for the gamma distribution. Consensus trees were calculated with Consense [41]. At last, phylogenetic trees were visualized with MEGA 3.1 [43]. In the tree figures shown, the topology support values are labeled on the Bayesian consensus tree in the order % Bayesian posterior probability/% bootstrap Maximum Likelihood to reduce and standardize the characters and figures used.

### Molecular modeling and tree construction on structural information

To create 3D models of the GK and the GK-HOOK-SH3 super domain of the different MAGUK subfamilies we used sequences of human origin. The BLAST E-value limit was set at 1.0e^-6 ^while template identification searches were performed, selecting for the best template through the SWISS-MODEL workspace [44,45]. For model building, refinement and visualization of the superimpositions Swiss-PdbViewer version 3.7 was used [44]. Used templates and their expect values are given in Additional file 2.

## Authors' contributions

CB conceived and designed the experiments. CB, AT and JA performed the experiments, while both CB and AT analyzed the data. AT and CB contributed materials/analysis tools and wrote the manuscript.

**Table 1:** The human MAGUK family proteins, their synonyms and chromosomal location

**Name**	**Synonyms**	**Acc. No. *H. sapiens***	**Chromosomal location *H. sapiens***
DLG-1	SAP-97	NM_004087	Chromosome 3
DLG-2	PSD-93, Chapsin-110	NM_001364	Chromosome 11
DLG-3	SAP-102, MRX-90	NM_021120	Chromosome X
DLG-4	PSD95, SAP-90	NM_001365	Chromosome 17
DLG-5	P-DLG	NP_004738	Chromosome 10
MPP-1	P55-1, EMP55, MRG1, AAG12	NM_002436	Chromosome X
MPP-2	P55-2	NM_005374	Chromosome 17
MPP-3	P55-3	NM_001932	Chromosome 17
MPP-4	P55-4, ALS2CR5, DLG6	NM_033066	Chromosome 2
MPP-5	P55-5, PALS1, stardust (Dm)	NM_022474	Chromosome 14
MPP-6	P55-6, PALS2, VAM1	NM_016447	Chromosome 7
MPP-7	P55-7, skf (Dm)	BC038105	Chromosome 10
ZO-1	TJP-1, tamou (Dm)	NM_175610	Chromosome 15
ZO-2	TJP-2	NM_004817	Chromosome 9
ZO-3	TJP-3	NM_014428	Chromosome 19
Carma1	CARD11, BIMP3	NM_032415	Chromosome 7
Carma2	CARD14, BIMP2	AF322642	Chromosome 17
Carma3	CARD10, BIMP1	AY028896	Chromosome 22
CASK	LIN2, camguk (Dm)	NM_003688	Chromosome X*
MAGI1	AIP3, WWP3, BAP-1	NM_001033057	Chromosome 3
MAGI2	AIP1	AF038563	Chromosome 7
MAGI3	AIP2, WWP2	AF038563	Chromosome 16

* CASK has a pseudogene on chromosome Y; (Dm = *Drosophila melanogaster*: name used in the fruitfly)

## Supplementary Material

Additional file 1 Protein domain dataset. Excel file that contains all sequences used and their corresponding accession numbers. Click here for file

Additional file 3 Phylogenetic analysis of the GK domain. Bayesian consensus trees including posterior probability values and bootstrap numbers for Maximum likelihood analysis of the GK domain. Click here for file

Additional file 2 Molecular Modeling. Sequences and templates used for molecular modeling including E-values. Click here for file

Additional file 5 Phylogenetic analysis of the PDZ domain. Bayesian consensus trees including posterior probability values and bootstrap numbers for Maximum likelihood analysis of the PDZ domain. Click here for file

Additional file 4 Phylogenetic analysis of the SH3 domain. Bayesian consensus trees including posterior probability values and bootstrap numbers for Maximum likelihood analysis of the SH3 domain. Click here for file

## References

[B1] Funke L, Dakoji S, Bredt DS (2005). Membrane-associated Guanylate Kinases Regulate Adhesion and Plasticity at Cell Junctions. Annual Review of Biochemistry.

[B2] González-Mariscal L., Betanzos A., Ávila-Flores A. (2002). MAGUK proteins: structure and role in the tight junction. Seminars in Cell & Developmental Biology.

[B3] Mburu P, Kikkawa Y, Townsend S, Romero R, Yonekawa H, Brown SDM (2006). Whirlin complexes with p55 at the stereocilia tip during hair cell development. PNAS.

[B4] Elias EM, Funke L, Stein V, Grant SG, Bredt DS, Nicoll RA (2006). Synapse-Specific and Developmentally Regulated Targeting of AMPA Receptors by a Family of MAGUK Scaffolding Proteins. Neuron.

[B5] Kornau HC, Schenker LT, Kennedy MB, Seeburg PH (1995). Domain interaction between NMDA receptor subunits and the postsynaptic density protein PSD-95. Science.

[B6] Hoover KB, Liao SY, Bryant PJ (1998). Loss of the Tight Junction MAGUK ZO-1 in Breast Cancer : Relationship to Glandular Differentiation and Loss ofHeterozygosity. Am J Pathol.

[B7] Pomerantz JL, Denny EM, Baltimore D (2002). CARD11 mediates factor-specific activation of NF-κB by the T cell receptor complex. EMBO Journal.

[B8] Nakamura S, Nakamura S, Matsumoto T, Yada S, Hirahashi M, Suekane H, Yao T, Goda K, Lida M (2005). Overexpression of caspase recruitment domain (CARD) membrane-associated guanylate kinase 1 (CARMA1) and CARD9 in primary gastric B-cell lymphoma. Cancer.

[B9] Kim E, Niethammer M, Rothschild A, Nung Jan Y, Sheng M (1995). Clustering of Shaker-type K+ channels by interaction with a family of membrane-associated guanylate kinases. Nature.

[B10] Zhang Y, Yeh S, Appleton BA, Held HA, Kausalya PJ, Phua DCY, Lee Wong W, Lasky LA, Wiesmann C, Hunziker W, Sidhu SS (2006). Convergent and Divergent Ligand Specificity among PDZ Domains of the LAP and Zonula Occludens (ZO) Families. J Biol Chem.

[B11] Chen VC, Li X, Perreault H, Nagy JI (2006). Interaction of Zonula Occludens-1 (ZO-1) with alpa-Actinin-4: Application of Functional Proteomics for Identification of PDZ Domain-Associated Proteins. J Proteome Res.

[B12] Kim E, DeMarco SJ, Marfatia SM, Chishti AH, Sheng M, Strehler EE (1998). Plasma Membrane Ca2+ ATPase Isoform 4b Binds to Membrane-associated Guanylate Kinase (MAGUK) Proteins via Their PDZ (PSD-95/Dlg/ZO-1) Domains. J Biol Chem.

[B13] Olsen O, Bredt DS (2003). Functional Analysis of the Nucleotide Binding Domain of Membrane-associated Guanylate Kinases. J Biol Chem.

[B14] Tavares GA, Panepucci EH, Brunger AT (2001). Structural Characterization of the Intramolecular Interaction between the SH3 and Guanylate Kinase Domains of PSD-95. Molecular Cell.

[B15] McGee AW, Bredt DS (1999). Identification of an Intramolecular Interaction between the SH3 and Guanylate Kinase Domains of PSD-95. J Biol Chem.

[B16] Takahashi SX, Miriyala J, Colecraft HM (2004). Membrane-associated guanylate kinase-like properties of {beta}-subunits required for modulation of voltage-dependent Ca2+ channels. PNAS.

[B17] Firestein BL, Rongo C (2001). DLG-1 Is a MAGUK Similar to SAP97 and Is Required for Adherens Junction Formation. Mol Biol Cell.

[B18] Jelen F, Oleksy A, Smietana K, Otlewski J (2003). PDZ domains - common players in the cell signaling. Acta Biochim Pol.

[B19] Fei K, Yan L, Zhang J, Sarras MP (2000). Molecular and biological characterization of a zonula occludens-1 homologue in Hydra vulgaris, named HZO-1. Development Genes and Evolution.

[B20] Adell T, Gamulin V, Perovic-Ottstadt S, Wiens M, Korzhev M, Müller IM, Müller WEG (2004). Evolution of Metazoan Cell Junction Proteins: The Scaffold Protein MAGI and the Transmembrane Receptor Tetraspanin in the Demosponge Suberites domuncula. Journal of Molecular Evolution.

[B21] Gough J, Karplus K, Hughey R, Chothia C (2001). Assignment of homology to genome sequences using a library of hidden Markov models that represent all proteins of known structure. Journal of Molecular Biology.

[B22] Amores A, Force A, Yan YL, Joly L, Amemiya C, Fritz A, Ho RK, Langeland J, Prince V, Wang YL, Westerfield M, Ekker M, Postlethwait JH (1998). Zebrafish hox Clusters and Vertebrate Genome Evolution. Science.

[B23] Corredor-Adamez M, Welten MCM, Spaink HP, Jeffery JE, Schoon RT, de Bakker MAG, Bagowski CP, Meijer AH, Verbeek FJ, Richardson MK (2005). Genomic annotation and transcriptome analysis of the zebrafish (Danio rerio) hox complex with description of a novel member, hoxb13a. Evolution & Development.

[B24] Willmon CL, Krabbenhoft E, Black ME (2006). A guanylate kinase//HSV-1 thymidine kinase fusion protein enhances prodrug-mediated cell killing. Gene Ther.

[B25] Lee S, Fan S, Makarova O, Straight S, Margolis B (2002). A Novel and Conserved Protein-Protein Interaction Domain of Mammalian Lin-2/CASK Binds and Recruits SAP97 to the Lateral Surface of Epithelia. Mol Cell Biol.

[B26] Blaszczyk J, Li Y, Yan H, Ji X (2001). Crystal structure of unligated guanylate kinase from yeast reveals GMP-induced conformational changes. Journal of Molecular Biology.

[B27] Stehle T, Schulz GE (1992). Refined structure of the complex between guanylate kinase and its substrate GMP at 2[middle dot]0 A resolution. Journal of Molecular Biology.

[B28] Dobrosotskaya I, Guy RK, James GL (1997). MAGI-1, a Membrane-associated Guanylate Kinase with a Unique Arrangement of Protein-Protein Interaction Domains. J Biol Chem.

[B29] Cavalier-Smith T, Chao EEY (2003). Phylogeny of Choanozoa, Apusozoa, and Other Protozoa and Early Eukaryote Megaevolution. Journal of Molecular Evolution.

[B30] Philippe H, Snell EA, Bapteste E, Lopez P, Holland PWH, Casane D (2004). Phylogenomics of Eukaryotes: Impact of Missing Data on Large Alignments. Mol Biol Evol.

[B31] King N, Hittinger CT, Carroll SB (2003). Evolution of Key Cell Signaling and Adhesion Protein Families Predates Animal Origins. Science.

[B32] King N (2004). The Unicellular Ancestry of Animal Development. Developmental Cell.

[B33] Ramesh MA, Malik SB, Logsdon JJM (2005). A Phylogenomic Inventory of Meiotic Genes: Evidence for Sex in Giardia and an Early Eukaryotic Origin of Meiosis. Current Biology.

[B34] Simpson AGB, Inagaki Y, Roger AJ (2006). Comprehensive Multigene Phylogenies of Excavate Protists Reveal the Evolutionary Positions of "Primitive" Eukaryotes. Mol Biol Evol.

[B35] Gupta RS, Aitken K, Falah M, Singh B (1994). Cloning of Giardia lamblia Heat Shock Protein HSP70 Homologs: Implications Regarding Origin of Eukaryotic Cells and of Endoplasmic Reticulum. PNAS.

[B36] Altschul SF, Madden TL, Schaffer AA, Zhang J, Zhang Z, Miller W, Lipman DJ (1997). Gapped BLAST and PSI-BLAST: a new generation of protein database search programs. Nucl Acids Res.

[B37] Letunic I, Copley RR, Pils B, Pinkert S, Schultz J, Bork P (2006). SMART 5: domains in the context of genomes and networks. Nucleic Acids Research.

[B38] Ponting CP, Schultz J, Milpetz F, Bork P (1999). SMART: identification and annotation of domains from signalling and extracellular protein sequences. Nucleic Acids Research.

[B39] Thompson JD, Gibson TJ, Plewniak F, Jeanmougin F, Higgins DG (1997). The CLUSTAL_X windows interface: flexible strategies for multiple sequence alignment aided by quality analysis tools. Nucleic Acids Research.

[B40] Huelsenbeck JP, Ronquist F (2001). MRBAYES: Bayesian inference of phylogenetic trees. Bioinformatics.

[B41] Felsenstein J (1989). PHYLIP -- Phylogeny Inference Package (Version 3.2). Cladistics.

[B42] Guindon S, Gascuel O (2003). A Simple, Fast, and Accurate Algorithm to Estimate Large Phylogenies by Maximum Likelihood. Systematic Biology.

[B43] Kumar S, Tamura K, Nei M (2004). MEGA3: Integrated Software for Molecular Evolutionary Genetics Analysis and Sequence Alignment. Briefings in Bioinformatics.

[B44] Schwede T, Kopp J, Guex N, Peitsch MC (2003). SWISS-MODEL: an automated protein homology-modeling server. Nucl Acids Res.

[B45] Kopp J, Schwede T (2006). The SWISS-MODEL Repository: new features and functionalities. Nucl Acids Res.

