# Supplementary figures and images for: Molecular evolution of the MAGUK family in metazoan genomes
Source: BMC Evol Biol. 2007 Aug 2;7:129. doi: 10.1186/1471-2148-7-129 (PMC1978500; doi:10.1186/1471-2148-7-129)

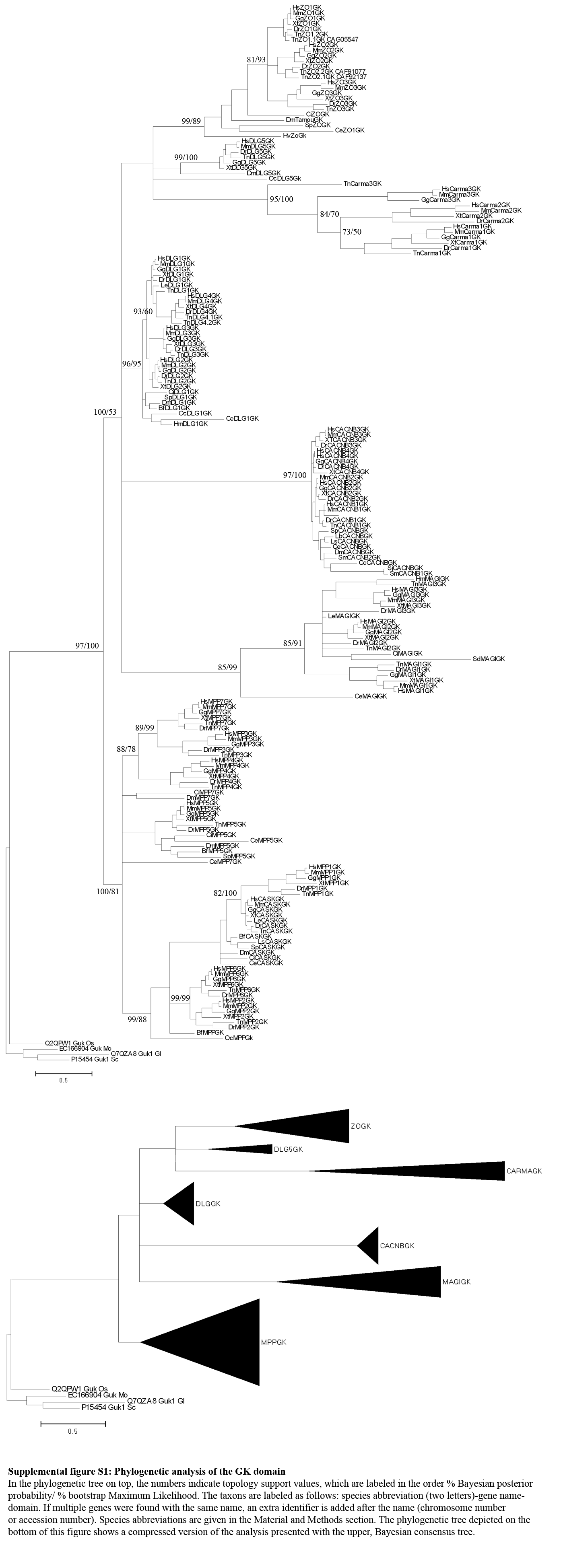

Supplement: Additional file 3 — Phylogenetic analysis of the GK domain. Bayesian consensus trees including posterior probability values and bootstrap numbers for Maximum likelihood analysis of the GK domain. [file 1471-2148-7-129-S3.tiff]

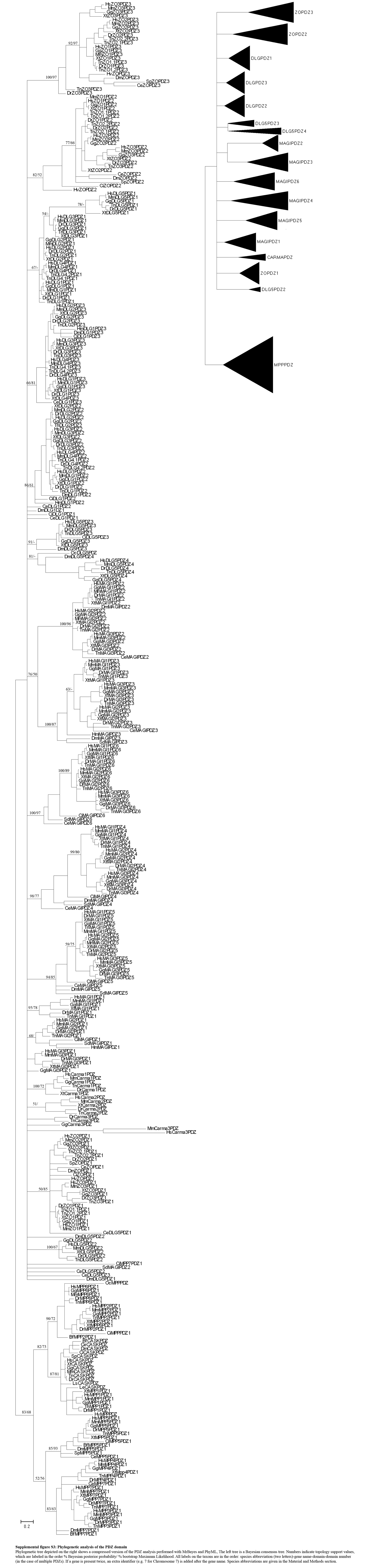

Supplement: Additional file 5 — Phylogenetic analysis of the PDZ domain. Bayesian consensus trees including posterior probability values and bootstrap numbers for Maximum likelihood analysis of the PDZ domain. [file 1471-2148-7-129-S5.tiff]

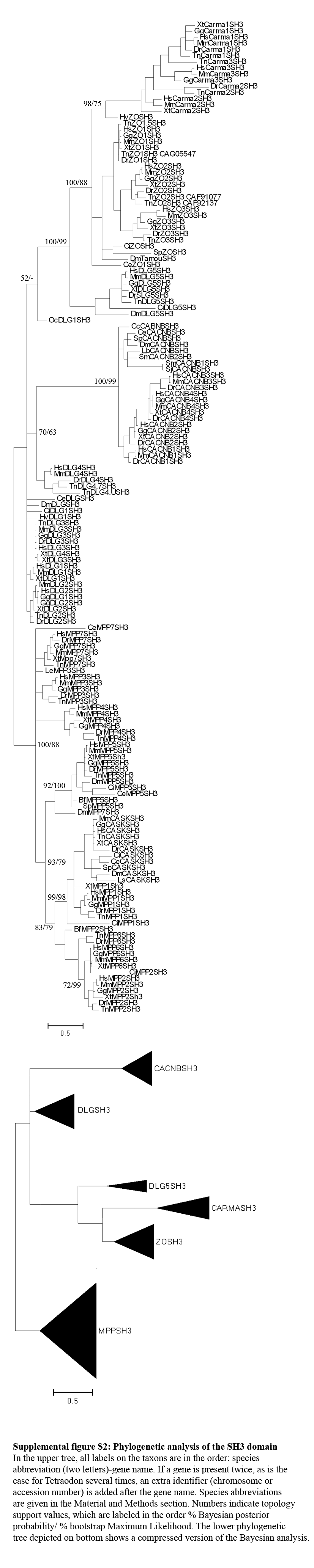

Supplement: Additional file 4 — Phylogenetic analysis of the SH3 domain. Bayesian consensus trees including posterior probability values and bootstrap numbers for Maximum likelihood analysis of the SH3 domain. [file 1471-2148-7-129-S4.tiff]
