# Supplementary material for: Molecular evolution of the MAGUK family in metazoan genomes
Source: BMC Evol Biol. 2007 Aug 2;7:129. doi: 10.1186/1471-2148-7-129 (PMC1978500; doi:10.1186/1471-2148-7-129)
Supplement: Additional file 2 — Molecular Modeling. Sequences and templates used for molecular modeling including E-values. [file 1471-2148-7-129-S2.doc]

| **Subfamily** | **Sequence *** | **PDB file** | **E-value** | **Domain** |
| --- | --- | --- | --- | --- |
| MPP | P70290 | 1kjwA | 1.83e-30 | SH3-GK |
| CACNB | Q02641 | 1t3IA | 4.24e-144 | SH3-GK |
| GMP Guk | Q16774 | 1lvgA | 2.59e-90 | GK |
| MPP | NM_022474 | 1lvgA | 1.65e-30 | MPP5 GK |
| CACNB | Q02641 | 1t3IA | 4.03e-91 | CACNB1 GK |
| DLG5 | NP_004738 | 1kjwA | 4.56e-23 | DLG5 GK |
| DLG | NM_004087 | 1kjwA | 3.66e-81 | DLG1 GK |
| ZO | NM_175610 | 1jxoA | 4.58e-9 | ZO1 GK |
| MAGI | NM_001033057 | 1ex7A | 1.43e-7 | MAGI1 GK |

* only human sequences were used

**List of species used and abbreviations:**

Species that were included in this dendogram shown in figure 3 are: **Bacteria:** *Gloeobacter violaceus*, *Thermotoga maritima*, *Clostridium tetani*, *Chlamydia muridarum*; Fungi: *Aspergillus fumigatus*, *Candida glabrata*, *Debaryomyces hansenii*, *Encephalitozoon cuniculi*, *Kluyveromyces lactis*, *Oryza sativa*, *Yarrowia lipolytica*; **Metazoa:** *Suberites domuncula, Oscarella carmela*, *Lymnaea stagnalis*, *Loligo bleekeri*, *Cyanea capillata*, *Hydra magnipapillata*, *Hydra vulgaris*, , *Schistosoma japonicum*, *Schistosoma mansoni, Caenorhabditis elegans, Drosophila melanogaster*, *Ciona intestinalis, Branchiostoma floridae, Leucoraja erinacea,Tetraodon nigroviridis*, *Danio rerio*, *Xenopus tropicalis, Gallus gallus*, *Bos taurus*, *Mus musculus*, *Homo sapiens*; Mycetozoa: *Dictyostelium discoideum*; Plantae: *Arabidopsis thaliana*, *Nicotiana tabacum*; **Protozoa:** *Giardia lamblia*, *Leishmania major*, *Monosiga ovata*, *Plasmodium yoelii yoelii*, *Tetrahymena thermophila*, *Trypanosoma cruzi*; **Viruses:** Vaccinia virus, Cowpox virus.
